# Psychiatric morbidity among undergraduate students of university of Ilorin, Kwara state, Nigeria

**DOI:** 10.1192/j.eurpsy.2021.342

**Published:** 2021-08-13

**Authors:** O. Buhari, A.J. Ogunmodede, O. Bolarinwa, O. Adegunloye, A. Adegoke, R. Oguntayo

**Affiliations:** 1 Dept Of Behavioural Sciences, UNIVERSITY OF ILORIN & University OF ILORIN TEACHING HOSPITAL, ILORIN, Nigeria; 2 Dept Of Behavioural Sciences, UNIVERSITY OF ILORIN TEACHING HOSPITAL, ILORIN, Nigeria; 3 Department Of Epidemiology And Community Health, Faculty Of Clinical Sciences, University of Ilorin/ University of Ilorin Teaching Hospital, ilorin, Nigeria; 4 Department Of Counseling Education, University of Ilorin, ilorin, Nigeria; 5 Department Of Psychology, university of Ilorin, Ilorin, Nigeria

**Keywords:** psychiatric morbidity, undergraduate students

## Abstract

**Introduction:**

The Nigerian tertiary education system admits mostly teenagers and young adults from different ethno-religious and family backgrounds, some of whom may have inherent risks and predisposition to mental illness. They then undergo stressful conditions related to the university life such as long durations of lectures, over-crowding, and lack of social amenities, haphazard lecture schedules as well as incessant industrial strike actions of academic and non- academic staff. In spite of these, there appears to be few studies on the burden of emotional and mental disorders among Nigerian University students, and none was cited suggesting interventions that may be appropriate.

**Objectives:**

The objectives of the study is to determine the prevalence of psychiatric morbidity and its associated factors among undergraduate students of Univesity of Ilorin

**Methods:**

This is a cross-sectional study using multi staged systematic randomization. A self-administered socidemographic questionnaire and the 12 item general health questionnaire (GHQ -12) was administered on 3,300 students.

**Results:**

Psychiatric morbidity was found to be 23.6% of the 3179 analyzable returned questionnaires. Factors found to be significantly associated with psychiatric morbidity included female gender, relationship with parents, parental employment status and family structure. Students on scholarship were more likely to have mental illness. Other associated factors include whether course of study was the preferred one and relationships with peers and lecturers on campus. About 46.6% of the students were willing to have internet based mental health intervention programmes.

**Conclusions:**

The data obtained from this study is relevant for the formation of mental health promotion and prevention programs on our campus.

**Disclosure:**

this study is part of the first phase of a three phase study. it aims to explore the factors associated with psychiatric morbidity among University student as a precursor for determining appropriate mental health interventions. it was partly funded by the

